# Previous tuberculosis modifies spirometry outcomes among small-scale gemstone miners in Tanzania: a cross-sectional, clinic-based study

**DOI:** 10.1136/bmjresp-2025-003490

**Published:** 2026-01-06

**Authors:** Florence J Mtei, Kassim Salim Msaji, Alexander W Mbuya, Stellah Mpagama, Patrick Howlett

**Affiliations:** 1 Kibong’oto Infectious Diseases Hospital, Kilimanjaro, Tanzania, United Republic of; 2 Nelson Mandela African Institute of Science and Technology, Arusha, Tanzania, United Republic of; 3 National Heart and Lung Institute, Imperial College London National Heart and Lung Institute, London, UK

**Keywords:** Occupational Lung Disease, Tuberculosis, Clinical Epidemiology

## Abstract

**Introduction:**

Small-scale miners are known to experience high silica exposures, associated with high rates of silicosis and Tuberculosis (TB). TB has been shown to worsen underlying impairment of lung function in miners. We describe the spirometry outcomes, according to previous TB status, among a large cohort of small-scale miners attending a screening centre.

**Methods:**

We collected cross-sectional spirometry and clinical data from consecutive miners and ex-miners, with negative Xpert TB results, attending a screening clinic in Northern Tanzania, between February 2018 and December 2020. Spirometry values assessed using the ATS/ERS 2019 quality criteria and compared with GLI 2022 global (GLIgl) reference values. We used multiple linear regression to model excess Forced Expiratory Volume in 1 s (FEV1) and Forced Vital Capacity (FVC) loss using an a priori interaction between duration of work and previous TB.

**Results:**

Of 542 participants with spirometry results, 80 (15%) reported previous TB. At least moderate (z-score ≤−2.5) FEV1 reductions were present in 51% of participants with previous TB and 18% of those without previous TB. For FVC, respective values were 34% and 10%. A miner with TB and 10 years of work was modelled to have lost 1405 (95% CI 1134 to 1676) mls of FEV1 and 1342 (95% CI 1042 to 1641) mls of FVC compared with GLIgl reference values. For miners without previous TB, the corresponding excess FEV1 and FVC losses were 693 (95% CI 581 to 804) mls and 624 (95% CI 504 to 743) mls, respectively.

**Discussion:**

Unmeasured silicosis may partially explain some of the observed effect of previous TB. However, this does not change our observation of a clinically significant burden of abnormal spirometry in a clinic-based population of small-scale miners. Reducing silica exposures and preventing TB are key to improving lung health in miners.

**WHAT IS ALREADY KNOWN ON THIS TOPIC**

- Very high silica exposures are associated with high prevalences of silicosis and tuberculosis (TB) among between 43 and 64 million small-scale miners globally.
- How occupational exposures and TB interact to influence spirometry values among small-scale miners is not described in the literature.

**WHAT THIS STUDY ADDS**

- We found that miners had lower median forced expiratory volume in 1 second (FEV1) and forced vital capacity values than predicted using the GLI global reference values. Those with a history of TB showed the greatest losses; for example, a miner with previous TB and 10 years of work was modelled to have lost 1405 (95% CI 1134 to 1676) mls of FEV1 compared with 693 (95% CI 581 to 804) mls in a miner with no history of TB.

**HOW THIS STUDY MIGHT AFFECT RESEARCH, PRACTICE OR POLICY**

- Reducing silica exposures and preventing TB are key measures to improve lung health in miners.

## Introduction

Among miners globally, multiple factors lead to an increased risk of impaired lung function compared with the general population.^1^ In 2013, the US Occupational Safety and Health Administration (OSHA) concluded that there is an ‘exposure-response relationship between exposure to respirable crystalline silica and the development of impaired lung function’.^2^ The extent of this impairment is unclear. One study among South African gold miners estimated that a miner with 0.3 mg/m^3^ dust exposure over a 24-year working lifetime would lose between 134 and 337 mls of Forced Expiratory Volume in 1 s (FEV1) and 110 and 324 mls of Forced Vital Capacity (FVC).^3^ When adjusted for silica exposure, evidence regarding the additional effect of silicosis is conflicting.^4 5^ Smoking rates among mining populations are often high;^1^ OSHA concludes its effect is either additive or potentially synergistic to that of silica exposure.^2^

The risk of tuberculosis (TB) also increases in a dose-response effect with silica exposure and consequently leads to impaired spirometry values.^6 7^ Among two studies of South African gold miners, a single TB episode was associated with 153 and 347 mls of excess FEV1 loss and 97 and 264 mls excess FVC loss, respectively;^8 9^ a further longitudinal study demonstrated accelerated FEV1 and FVC decline following TB diagnosis.^10^ Although all studies assumed a fixed effect of TB, it is possible that the effect of TB may vary according to cumulative silica exposures.

There are estimated to be between 43 and 64 million small-scale miners worldwide^11^ who are exposed to average silica intensities that range between 4 and 2500 times above the US OSHA permissible time-weighted average exposure limit of 0.05 mg/m^3^.^1 12^ Prevalences of silicosis and TB are accordingly high. Much-needed studies of spirometry outcomes, particularly in relation to post-TB lung disease, are limited in this population.^1 13 14^

The occupational health screening centre at Kibong’oto Infectious Diseases Hospital (KIDH), Northern Tanzania, provides screening for silicosis and TB to miners and ex-miners. Almost all miners are from the Mirerani ward (commonly referred to as Mererani), a gemstone mining area located approximately 50 km from KIDH which has a workforce of approximately 12 000 active, small-scale miners.

We aimed to describe the spirometry outcomes, according to previous TB status, of a large cohort of small-scale miners attending the occupational health service centre (OHCS) at KIDH. We investigated the association between flexibly modelled work duration and FEV1 and FVC change, allowing for effect modification by previous TB disease.

## Method

### Study design and setting

This cross-sectional study uses routinely collected proforma data from consecutive outpatient miners attending the OHSC between February 2018 and December 2020.

### Participants

Inclusion criteria were current miners and ex-miners aged >16 years of age. During the study period, spirometry was offered routinely, free of charge, for all participants without contraindications attending the OHSC in whom TB was excluded by GeneXpert MTB-RIF (Cepheid) testing. Exclusion criteria were patients with no documented previous TB status or no spirometry results.

### Spirometry testing

Spirometry was conducted at the OHSC in a dedicated room by trained staff. Syringe calibration was performed prior to each clinical session. Height and weight were measured with no shoes prior to testing. FEV1 and FVC were measured using a spirometer (EasyOn PC, ndd) in the sitting position without a nose clip. Participants were coached to completion of three acceptable manoeuvres or a maximum of 8 attempts. Contemporaneous acceptability and repeatability were assessed by trained study staff and by the spirometry software (EasyOne Connect, ndd) using the ATS/ERS 2005 criteria, which provides a single summary grade for FEV1 and FVC.^15 16^ For the purposes of our analysis, a single respiratory physician (PH) later re-assessed acceptability and repeatability using the updated ATS/ERS 2019 criteria, which grades the FEV1 and FVC separately.^17^ Thus, FEV1 *or* FVC values meeting A to C criteria were retained for our primary analyses; ratios were included where both FEV1 *and* FVC values met criteria. Sensitivity analysis included only A values.

Our primary analysis used prediction values from the Global Lung Initiative 2022, race neutral, global equation (GLIgl).^18^ Severity was classified using z-scores: values greater than −1.645 were considered normal, between −2.5 and −1.645 indicated mild impairment, between −4<and ≤ −2.5 indicated moderate impairment and less than −4 indicated severe impairment. Excess loss was defined as the difference between the predicted and observed value; a positive difference denotes an observed volume *less* than predicted. Reference values were also calculated using National Health and Nutrition Examination Survey (NHANES III)^19^ and 1993 GLI reference equations,^20^ both using African-American as the ethnicity variable. As per the ERS/ATS technical standard,^21^ normal lung function was defined as a normal FEV₁/FVC ratio (z > –1.645) and a normal FVC (z > –1.645), restriction as a normal FEV₁/FVC ratio (z > –1.645) and a reduced FVC (z ≤ –1.645), obstruction as a reduced FEV₁/FVC ratio (z ≤ –1.645) and normal FVC (z > –1.645) and mixed as a reduced FEV₁/FVC ratio (z ≤ –1.645) and reduced FVC (z ≤ –1.645).

### Other variables

Clinic data was collected on a paper proforma then entered to an Excel database. Previous TB and current tobacco smoking was measured as a binary variable based on self-report. HIV testing is routinely performed if the participant is not already linked to treatment or has not tested in the past 3 months.

### Sample size

Study size was limited by the availability of retrospective data. Nevertheless, using our sample size of 80 miners and ex-miners with previous TB and 462 without previous TB and assumptions of: a SD of 1 L in FEV1 and FVC values, a normal approximation for the z-statistic, a two-tailed α of 0.05 and β of 0.2, we were powered to detect a mean difference of 339 mls between the groups.

### Statistical methods

Participant characteristics and spirometry outcome values are summarised categorically or with median and interquartile values (IQR), as appropriate. Our a priori linear regression model of excess loss included age, miner or ex-miner status, HIV status, smoking status, and the duration of work and previous TB as interaction terms. Duration was modelled flexibly using a restricted cubic spline with four knots at the 5th, 35th, 65th and 95th centiles. For both FEV1 and FVC, this equated to 2, 7, 15 and 26 years. To observe the interaction between previous TB and duration of work, predicted spirometry values are presented in graphical and tabular form with 95% CIs, with all other variables held at their respective mode (categorical variables) or mean (continuous variables) values. Other model variables are not presented in the main text.^22^ We performed a complete cases analysis as very few data were missing. All analyses are performed using R V.4.3.2, including the use of ‘rspiro’, ‘rms’ and ‘marginaleffects’ packages. All code is available at https://github.com/pjhowlett/ssm_clin_spiro.

### Ethical clearance

Ethical clearance to analyse routinely collected clinical data was granted from the Joint Ethics and Review Committee of KIDH, the Nelson Mandela Institution of Science and Technology, and the Centre for Educational Development in Health, Arusha with registration number KNCHREC/001 (April 2021).

### Funding

For a period between February 2018 and December 2020, investigations were provided for free to miners and ex-miners through the Southern African Development Community, Tuberculosis in the Mining Sector in Southern Africa programme.^23^ One author (PH) was supported by an MRC Clinical Research Training Fellowship (MR/W024861/1). Neither funder had any involvement in the design, analysis or writing of this study.

### Patient and public involvement

The OHSC was initially designed with significant involvement from miners and, in particular, with an organisation of ex-TB patients (MKUTA). The organisation of Ex-TB patients provides outreach to the mining communities, referring patients to the service and providing education to miners and their families. The results from this study were presented at an engagement workshop in September 2025, with attendance from a wide range of stakeholders, from miners and community members to government health and mining services officials. From this meeting, a joint policy brief, drafted and signed by all attendees, has been shared nationally and with high-level policy makers.

## Results

### Patient characteristics

From a total of 622 miners and ex-miners in the clinic, 27 (4) were excluded as they had no spirometry results and two (0%) as no previous TB status recorded (online supplemental figure 1). Of the remaining 593 participants, 542 (92%) had either FEV1 or FVC manoeuvres that met quality criteria. These included 503 (78%) FEV1 and 505 (85%) FVC results that met quality criteria according to ATS/ERS 2019 quality criteria. Quality criteria for *both* FEV1 and FVC results were met for 476 (80%) participants. Online supplemental table 1 summarises quality scores compared with the single summary grading, based on the 2005 ATS/ERS guidance, that was provided by the software.

Of 542 participants who met either FEV1 or FVC quality criteria, almost all (537/542, 99%) chest participants were male, most (426/534, 79%) were current miners and 80 (15%) reported having previously had TB (table 1). The median age of participants with previous TB was slightly higher than those with no history of TB; 45.0 years (IQR 40 to 50 years) compared with 41 years (IQR 36 to 48 years). Similarly, the median number of years worked in mining was slightly higher among those with previous TB; 15 years (IQR 10 to 20 years) compared with 10 years (5 to 16 years). According to body mass index (BMI), more participants with previous TB were underweight (< 18.5 kg/m^2^); 18/80 (23%) compared with 44/458 (10%). HIV prevalence was similar among those with previous TB (4/79, 5.1%) compared with those without previous TB (20/454, 4.4%); nine participants had a missing HIV status. Being a current person who smokes was common, with a higher percentage among participants with previous TB (18/78, 23%) than those without previous TB (75/459, 16%). As expected among clinic patients, patients experienced a significant symptom burden; for example, 375/542 (69%) reported a cough of any duration (online supplemental table 2). The prevalences of symptoms were broadly similar between groups, except for haemoptysis which was reported by 7/80 (8.9%) of those with previous TB and 21/440 (4.6%) of those with no previous TB.

**Table 1:** Characteristics of miners and ex-miners attending the Occupational Health Service Centre at Kibong’oto Infectious Diseases Hospital, Tanzania, according to previous TB status

	Previous TB (n=80)	No previous TB (n=462)	All participants (n=542)
Age category			
16–39	19 (23.8%)	185 (40.0%)	204 (37.6%)
40–59	57 (71.2%)	258 (55.8%)	315 (58.1%)
>60	4 (5.0%)	19 (4.1%)	23 (4.2%)
N-Miss	0	0	0
Gender			
Female	1 (1.2%)	4 (0.9%)	5 (0.9%)
Male	79 (98.8%)	458 (99.1%)	537 (99.1%)
N-Miss	0	0	0
Years worked in mining			
Median (Q1, Q3)	15.0 (10.0, 20.0)	10.0 (5.0, 16.0)	10.0 (5.0, 17.0)
N-Miss	0	1	1
Years worked in mining category			
<10	15 (18.8%)	211 (45.8%)	226 (41.8%)
10–19	44 (55.0%)	170 (36.9%)	214 (39.6%)
>20	21 (26.2%)	80 (17.4%)	101 (18.7%)
N-Miss	0	1	1
BMI category (kg/m^2^)			
Underweight (<18.5)	18 (22.5%)	44 (9.6%)	62 (11.5%)
Normal (18.5–24.9)	53 (66.2%)	313 (68.3%)	366 (68.0%)
Overweight/obese (>25)	9 (11.2%)	101 (22.1%)	110 (20.4%)
N-Miss	0	4	4
Occupation			
Current miner	51 (63.8%)	375 (81.2%)	426 (78.6%)
Ex-miner	29 (36.2%)	87 (18.8%)	116 (21.4%)
N-Miss	0	0	0
HIV status			
Negative	75 (94.9%)	434 (95.6%)	509 (95.5%)
Positive	4 (5.1%)	20 (4.4%)	24 (4.5%)
N-Miss	1	8	9
Tobacco people who smoke			
No	60 (76.9%)	384 (83.7%)	444 (82.7%)
Yes	18 (23.1%)	75 (16.3%)	93 (17.3%)
N-Miss	2	3	5

BMI, Body Mass Index; HIV, HIV Immunodeficiency Virus; TB, Tuberculosis.

### Spirometry results

Abnormal spirometry values were common among both groups, and those with previous TB had lower raw and adjusted spirometry values than those with no history of TB (table 2). Among those with a previous history of TB, 49/68 (72%) had abnormal spirometry; the most common pattern was restrictive (24/68, 35%), followed by mixed (20/68, 29%) and obstructive (5/68, 7.4%) patterns. Among those with no history of TB, 146/408 (36%) had abnormal spirometry, with the most common pattern being restrictive (75/408, 18%) followed by mixed (37/408, 9.1%) and obstructive (34/408, 8.3%).

**Table 2:** Spirometry outcomes for miners and ex-miners attending the Occupational Health Service Centre at KIDH, Tanzania, according to previous TB status

	Previous TB	No previous TB	All participants
Measured FEV1/mls			
Median (Q1, Q3)	2032 (1491, 2582)	2815 (2302, 3269)	2734 (2146, 3199)
Excess loss FEV1/mls (GLIgl)			
Median (Q1, Q3)	1344 (768, 1839)	602 (286, 1061)	677 (331, 1195)
Excess loss FEV1/mls (NHANES)			
Median (Q1, Q3)	980 (465, 1515)	284 (-35, 711)	355 (37, 872)
FEV1 z-score category (GLIgl)			
Total	75 (100%)	438 (100%)	513 (100%)
Normal	21 (28.0%)	281 (64.4%)	302 (59.1%)
Mild	16 (21.3%)	78 (17.9%)	94 (18.4%)
Moderate	27 (36.0%)	70 (16.1%)	97 (19.0%)
Severe	11 (14.7%)	7 (1.6%)	18 (3.5%)
Percent predicted FEV1 (%) (GLIgl)			
Median (Q1, Q3)	62 (47, 77)	83 (69, 92)	81 (65, 90)
Measured FVC/mls			
Median (Q1, Q3)	2873 (2371, 3330)	3575 (3087, 4019)	3484 (2908, 3989)
Excess loss FVC/mls (GLIgl)			
Median (Q1, Q3)	1150 (709, 1966)	546 (250, 1011)	590 (277, 1129)
Excess loss FVC/mls (NHANES)			
Median (Q1, Q3)	796 (296, 1546)	180 (-131, 632)	230 (-83, 743)
FVC z-score category (GLIgl)			
Total	73 (100%)	432 (100%)	505 (100%
Normal*	27 (37.0%)	315 (73.3%)	342 (68.0%)
Mild	21 (28.8%)	71 (16.5%)	92 (18.3%)
Moderate	20 (27.4%)	38 (8.8%)	58 (11.5%)
Severe	5 (6.8%)	6 (1.4%)	11 (2.2%)
Percent predicted FVC (%) (GLIgl)			
Median (Q1, Q3)	71 (57, 84)	87 (76, 94)	86 (73, 93)
FEV1/FVC ratio (%)			
Median (Q1, Q3)	73 (65, 81)	80 (73, 84)	79 (72, 84)
Spirometry category (GLIgl)			
Total	68 (100%)	408 (100%)	478 (100%
Normal	19 (27.9%)	262 (64.2%)	281 (59.0%)
Obstructive	5 (7.4%)	34 (8.3%)	39 (8.2%)
Restrictive	24 (35.3%)	75 (18.4%)	99 (20.8%)
Mixed	20 (29.4%)	37 (9.1%)	57 (12.0%)

Z-score category: >−1.645 normal, between −1.65 and −2.5 mild impairment, between −2.5 and −4 moderate impairment, and <−4 severe impairment. Total participants for FEV1, FVC and ratio/category values 511, 503 and 476, respectively.

* This group includes 17 participants with mild FEV1 loss, who would be classified as preserved ratio impaired spirometry (PRISm), defined as FEV₁ z-score ≤ –1.645 with FEV₁/FVC z-score > –1.645.

FEV1, Forced Expiratory Volume in one second; FVC, Forced Vital Capacity; GLIgl, GLI global reference values; NHANES III, Third National Health and Nutrition Examination Survey, USA; TB, Tuberculosis.

**Figure 1:**
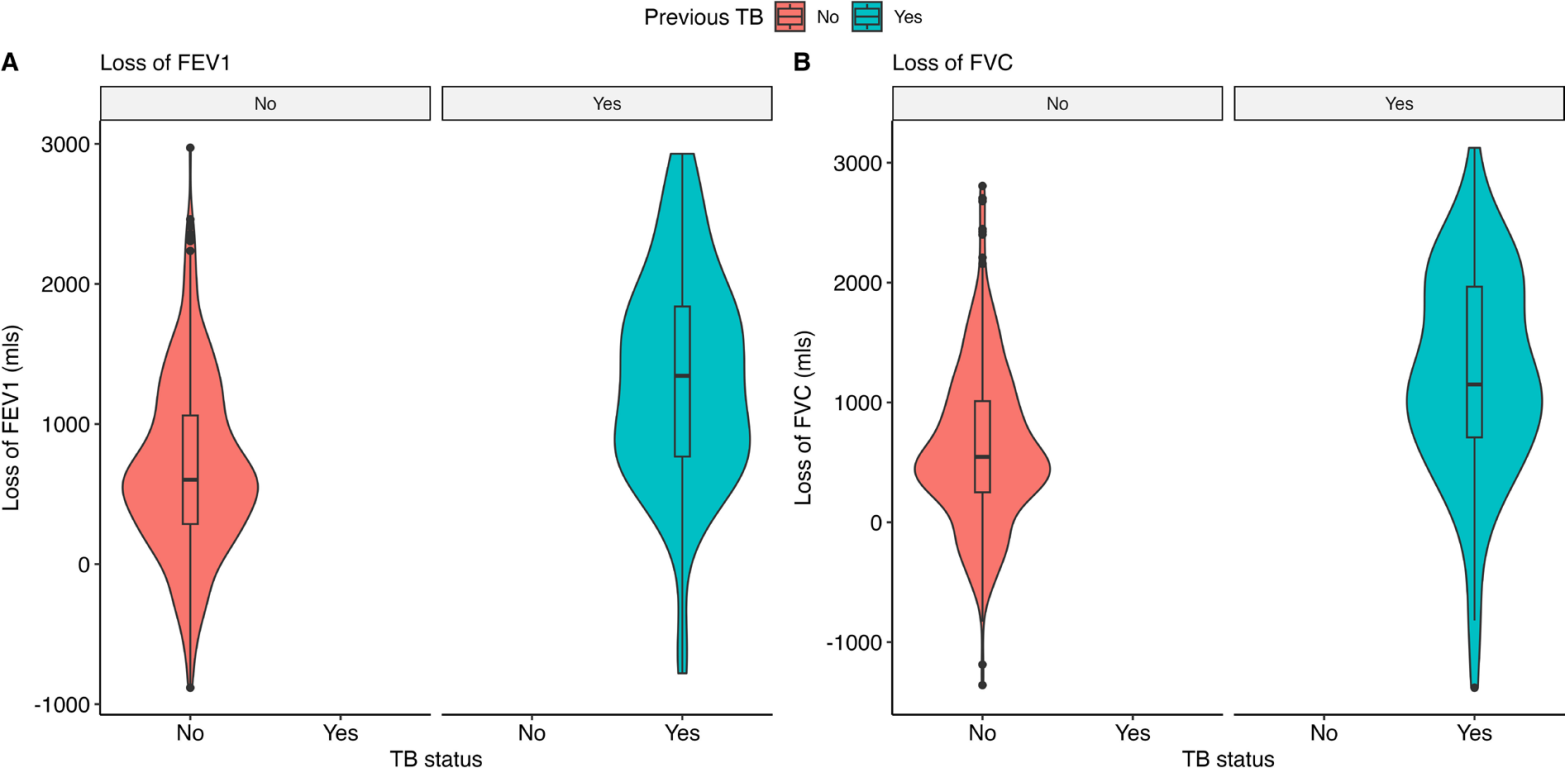
A violin plot overlayed onto a boxplot of excess loss in mls of (**A**) FEV1 and (**B**) FVC among current and ex-miners attending the Occupational Health Service Centre at Kibong’oto Infectious Diseases Hospital, Tanzania, estimated using the GLIgl equations. FEV1, Forced Expiratory Volume in one second; FVC, Forced Vital Capacity. Loss of FEV1; No previous TB n=436, Previous TB n=75. Loss of FVC; No previous TB n=430, Previous TB n=73. TB, Tuberculosis.

These abnormalities correspond to a relatively high severity of disease (table 2 and figure 1). Among miners with previous TB, 38/75 (51%) had at least moderate impairment of FEV1 (based on z-score) and 25/73 (34%) had at least moderate impairment of FVC. Among miners with no history of TB, these figures were lower at 77/436 (18%) and 44/430 (10%), respectively. Among miners with a history of TB, 54/75 (72%) and 46/73 (63%) had FEV1 and FVC values below the lower limit of normal (LLN), respectively. In these ‘below lower limit of normal’ groups, excess losses below the LLN were large (FEV1: median 816 mls, IQR 407 to 1172 mls below LLN; FVC: median 700 mls, IQR 287 to 1153 mls below LLN). Among those with no history of TB, 155/436 (36%) and 115/430 (27%) had FEV1 and FVC values below the LLN, respectively. Below LLN, losses were less than the previous TB group (FEV1: median 431 mls, IQR 180 to 736 mls below LLN; FVC: median 354 mls, IQR 174 to 676 mls below LLN).

**Figure 2:**
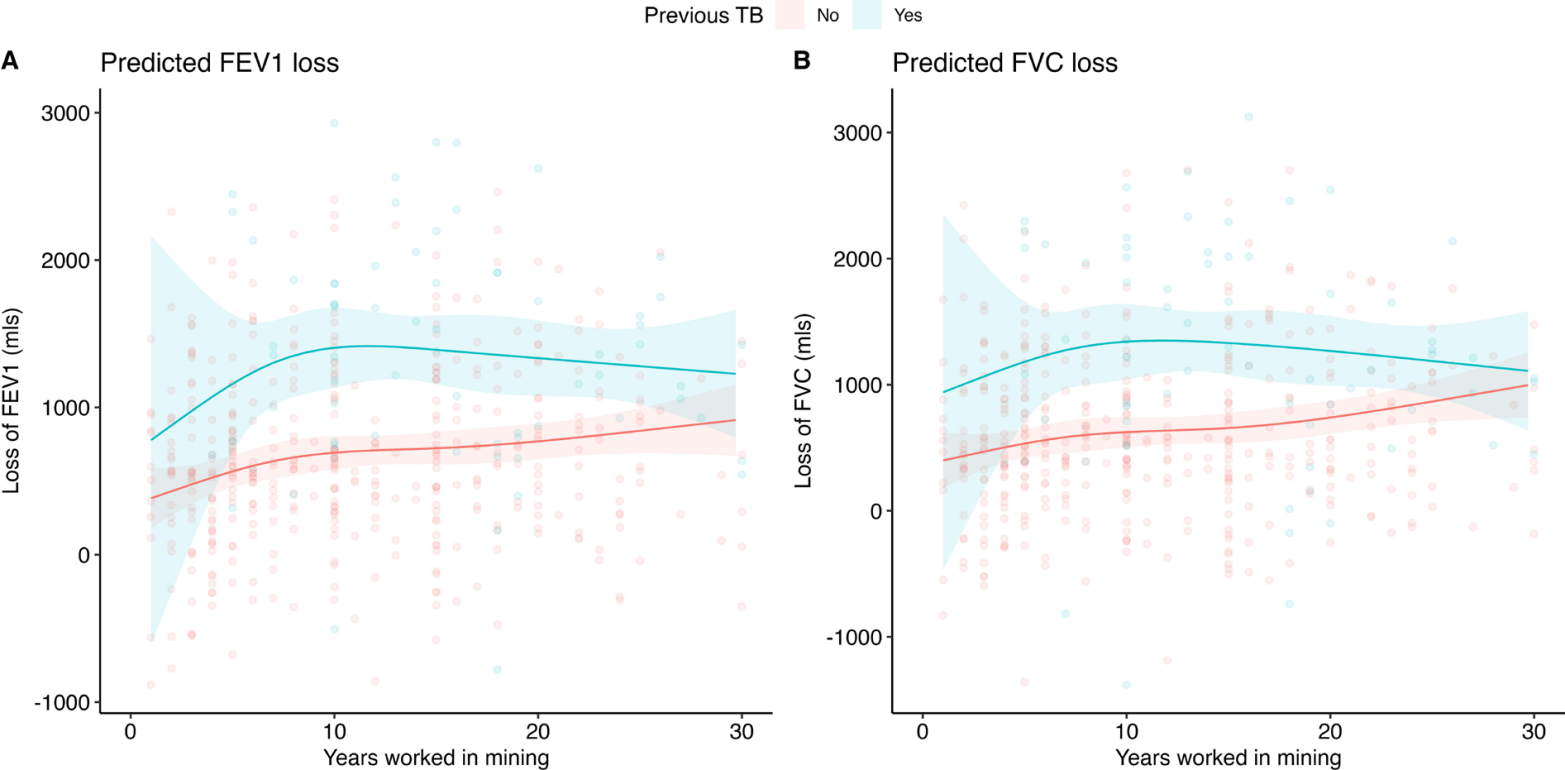
Predicted loss in mls of (**A**) FEV1 and (**B**) FVC in a male, non-smoker, HIV-negative, current miner, aged 42 years, with and without previous TB, according to a linear regression model. The duration of mining is modelled using a restricted cubic spline. The line graph represents a linear regression model with 95% CIs, individual points represent estimates of participants. FEV1, Forced Expiratory Volume in one second; FVC, Forced Vital Capacity; TB, Tuberculosis.

Among participants with previous TB, median FEV1 loss was 1344 mls (IQR 768 to 1839 mls) and median FVC loss was 1150 mls (IQR 709 to 1966 mls) (table 2 and figure 2). Among participants with no history of TB, median FEV1 loss was 602 mls (IQR 286 to 1061 mls) and median FVC loss was 546 mls (IQR 250 to 1011 mls). When NHANES III and GLI 2011 with ethnicity adjustment were used for reference, the z-score proportions and excess loss of spirometry values were reduced (table 2 and online supplemental table 3). Our sensitivity analysis using only A grade spirometry did not significantly alter our findings (online supplemental table 4).

### Modelling outcomes

Using GLI global predicted values to estimate the outcome of excess loss in our linear regression model, a male non-smoker, HIV-negative, current miner, aged 42 years who has a history of previous TB was predicted to lose 1160 mls (95% CI 690 to 1630 mls) FEV1 after 5 years of work (figure 2 and table 3). This rose to 1405 mls (95% CI 1134 to 1676 mls) after 10 years’ work, after which there was a slight decrease at 20–30 years. A miner with the same characteristics, but no history of previous TB, was predicted to lose 566 mls (95% CI 460 to 671 mls) of FEV1 after 5 years before excess loss steadily increased over the number of years of work, to 770 mls (95% CI 649 to 891 mls) at 20 years. A similar pattern is observed for FVC values, although with lower overall volume loss and an attenuated difference between the two groups. The same miner with a history of previous TB was predicted to lose 1182 mls (95% CI 710 to 1654 mls) of FVC after 5 years of work, rising to 1342 mls (95% CI 1042 to 1641 mls) after 10 years. If there was no history of TB, the miner was predicted to lose 533 mls (95% CI 421 to 645 mls) of FVC after 5 years before slowly increasing over the number of years of work exposure, to 739 mls (95% CI 609 to 870 mls) after 20 years. Our model was not constructed to assess the effect of other variables; however, their coefficients for both FEV1 and FVC are presented in online supplemental tables 5 and 6.

**Table 3:** Predicted FEV1 and FVC loss in mls of a male, non-smoker, HIV-negative, current miner, aged 42 years with and without previous TB, according to linear regression model

FEV1 (n=498)		
Duration of mining (years)	Predicted FEV1 in mls loss in miner with previous TB	Predicted FEV1 in mls loss in miner with no previous TB
1	778 (95% CI −610 to 2166)	383 (95% CI 182 to 585)
5	1160 (95% CI 690 to 1630)	566 (95% CI 460 to 671)
10	1405 (95% CI 1134 to 1676)	693 (95% CI 581 to 804)
20	1334 (95% CI 1079 to 1590)	770 (95% CI 649 to 891)
30	1225 (95% CI 777 to 1672)	919 (95% CI 669 to 1168)

FVC (n=488)		
Duration of mining (years)	Predicted FVC in mls loss in miner with previous TB	Predicted FVC in mls loss in miner with no previous TB
1	940 (95% CI −470 to 2350)	399 (95% CI 181 to 618)
5	1182 (95% CI 710 to 1654)	533 (95% CI 421 to 645)
10	1342 (95% CI 1042 to 1641)	624 (95% CI 504 to 743)
20	1267 (95% CI 992 to 1543)	739 (95% CI 609 to 870)
30	1105 (95% CI 617 to 1592)	1004 (95% CI 739 to 1269)

FEV1 and FVC loss outcomes were calculated using the GLIgl equations. Duration of mining is modelled using a restricted cubic spline.

FEV1, Forced Expiratory Volume in one second; FVC, Forced Vital Capacity; TB, Tuberculosis.

## Discussion

There is limited evidence regarding the effect of previous TB disease on spirometry outcomes among small-scale miners. Our cross-sectional, clinic-based study found that, when using the GLIgl 2022 reference values, a large proportion of miners had abnormal spirometry values. The prevalence of abnormality was higher and the severity more pronounced among miners with previous TB, compared with those without previous TB. At least moderate category FEV1 reductions were present in 51% of miners with previous TB and 18% of those without previous TB. At least moderate category FVC reductions were present in 34% of miners with previous TB and 10% of those without previous TB. Despite this, the most frequent spirometry pattern was a restrictive pattern, present among 35% of the miners with previous TB and 18% of those without previous TB, although mixed restriction-obstruction and obstruction were also common.

Our model of excess FEV1 and FVC loss demonstrated that, among both previous and no previous TB groups, excess loss increased with the number of years of mine working. As previously reported,^38^,^10^ previous TB was also associated with increased and clinically important loss of lung function. Silicosis was an important, unmeasured mediator between silica exposure and previous TB.^1 9^ Not being able to adjust for it means we cannot confidently attribute the observed spirometry difference between previous TB and no previous TB to previous TB alone. Silicosis has been shown to be common and inversely associated with work duration in our study population, potentially due to the health worker effect.^14^ Thus, the greater excess loss of spirometry in those with previous TB (compared with no previous TB) between 5 and 10 years of work may be explained by an unmeasured silicosis.^24^ Nevertheless, large excess losses, for example, 1405 mls of FEV1 and 1342 mls of FVC in an average miner with 10 years work duration and previous TB, emphasise the importance of TB prevention, including wider roll-out of preventive therapy^25^ and reduced silica exposure^26^; the latter being associated with a wider range of benefits. Potentially large effect sizes may make miners an interesting group for trials of novel, adjunctive TB therapies.^27^

Our clinic-based sample included older, self-presenting miners with longer work durations, more previous TB and higher smoking prevalences than our previous workforce-based study of rock drillers in Mererani, which demonstrated 12% with abnormal spirometry values.^14^ compared with three studies among South African gold miners,^9 10 28^ raw FEV1 and FVC values were lower and excess losses larger, despite our participants being younger and having a lower prevalence of previous TB. Furthermore, miners in our study with any previous TB had lower raw FEV1 and FVC values than miners with >3 episodes of TB in another study of South African gold miners.^8^ An explanation for the lower values is self-presentation of more symptomatic miners and ex-miners in our clinic population, compared with workforce studies. An additional reason for worse adjusted values is our choice of GLI global reference ranges which provide higher predicted values than models in previous studies. However, re-analysis with NHANES III or 2011 GLI ethnicity adjusted reference values still demonstrated a higher prevalence of abnormalities than previous studies. Importantly, greater loss at a workforce level remains plausible. As is common among small-scale miners,^1^ median recorded silica intensities in Mererani are very high; between 25 and 2440 times above the US OSHA permissible exposure level.^2629^,^31^

Our results demonstrated a large symptom burden. However, in contrast to previous evidence,^10^ those with previous TB did not appear more symptomatic; this may relate to self-presentation by symptomatic participants in both groups. Our experience, published case studies among miners and general population studies suggest silicosis and TB among miners are associated with significant morbidity and mortality and have a significant effect on livelihoods;^32^,^34^ reduced lung function may be a mediator or marker of this. That 1 in 5 participants with previous TB and 1 in 10 without previous TB who were underweight suggests nutrition may be a modifiable determinant.

Strengths of our study include a relatively large data set in the context of large yet underserved, global small-scale mining population^13^ and an explicit quality assessment approach to spirometry. However, we have several important study limitations. Causal inference is limited by lack of data on important variables, including silicosis status and number and date of previous TB episodes. Previous TB was self-reported; as silicosis and TB are commonly and mutually misclassified, the effect of this bias may be bidirectional and is unknown. Smoking status was self-reported as a binary variable of current or non-tobacco smoker; residual confounding may explain the lack of effect of smoking. BMI is likely associated with slightly reduced FEV1 and FVC in the highest and lowest groups.^35 36^ We did not adjust for BMI in our regression model as the effect is likely relatively small and, more importantly, the lower weight in those with previous TB is plausibly causally related to their previous TB. Importantly, however, nutrition may represent a therapeutic target in this group.^37^ The choice of when and which reference standard is most relevant is a matter of current debate.^38^ We chose GLI global values for our main analysis to represent the loss of lung function compared with an ideal predicted value. However, this may overestimate loss attributable to occupational exposures if predicted peaks are never attained due to life-course events that happened before starting work. That our baseline ‘intercept’ values among miners with ‘no’ years of work and no previous TB are below normal suggests predicted peaks may not have been reached. Although ethnicity-adjusted values have well-documented limitations,^39^ in this situation their use, at a population level, may better reflect loss attributable to occupation. Ideally, cohort studies are needed to measure longitudinal losses. Alternatively, identifying local controls may be challenging as young workers in physically active jobs may have supra-normal spirometry results compared with the general population. Finally, our study is not generalisable to the general small-scale mining population, but indicative of the self-presenting clinic population, including ex-miners.

## Conclusion

We demonstrate a significant burden of abnormal spirometry among small-scale gemstone miners that is associated with duration of work and previous TB. Given the between 43 and 64 million small-scale miners worldwide, better knowledge of spirometry outcomes is important. Ultimately, high silica exposure is the driving force to which a large proportion of reduced lung function is attributable. Interventions that reduce silica exposure are urgently needed.

## Supplementary material

10.1136/bmjresp-2025-003490 online supplemental file 1

## Data Availability

Data are available upon reasonable request.
