# Supplementary material for: Previous tuberculosis modifies spirometry outcomes among small-scale gemstone miners in Tanzania: a cross-sectional, clinic-based study
Source: BMJ Open Respir Res. 2026 Jan 6;13(1):e003490. doi: 10.1136/bmjresp-2025-003490 (PMC12778310; doi:10.1136/bmjresp-2025-003490)
Supplement: online supplemental file 1 [file bmjresp-13-1-s001.docx]

**Supplementary Material**

**Previous tuberculosis modifies spirometry outcomes among small-scale gemstone miners in Tanzania: A cross-sectional, clinic-based study**

Authors: Florence Jared Mtei^1^ MD, Kassim Msaji^1^ MD, Alexander William Mbuya^1^ MD, Stellah Mpagama^1,2^ PhD MD**,** Patrick Howlett MbChB MSc^3^

Table of Contents

[Supplementary Figure 1. Flow diagram of eligible participants and spirometry quality 2](#_Toc198825888)

[Supplementary Table 1. Comparison of manual quality assessment and as applied by spirometry software. 3](#_Toc198825889)

[Supplementary Table 2. Table of symptoms among miners and ex-miners attending the Occupational Health Screening Centre at KIDH, Tanzania, according to previous TB status 4](#_Toc198825890)

[Supplementary Table 3. Spirometry outcomes for miners and ex-miners attending the Occupational Health Screening Centre at KIDH, Tanzania, according to previous TB status, using NHANES III and 2011 GLI reference ranges, adjusted for ethnicity. 5](#_Toc198825891)

[Supplementary Table 4. Sensitivity analysis of Spirometry outcomes for miners and ex-miners attending the Occupational Health Screening Centre at KIDH, Tanzania, according to previous TB status, restricting to only A grade spirometry 6](#_Toc198825892)

[Supplementary Table 5. Coefficients of variables included in the model of outcome of FEV1 with the exposure of number of years of mine work and previous TB, included as an interaction term 7](#_Toc198825893)

[Supplementary Table 6. Coefficients of variables included in the model of outcome of FVC with the exposure of number of years of mine work and previous TB, included as an interaction term 8](#_Toc198825894)

# Supplementary Figure 1. Flow diagram of eligible participants and spirometry quality


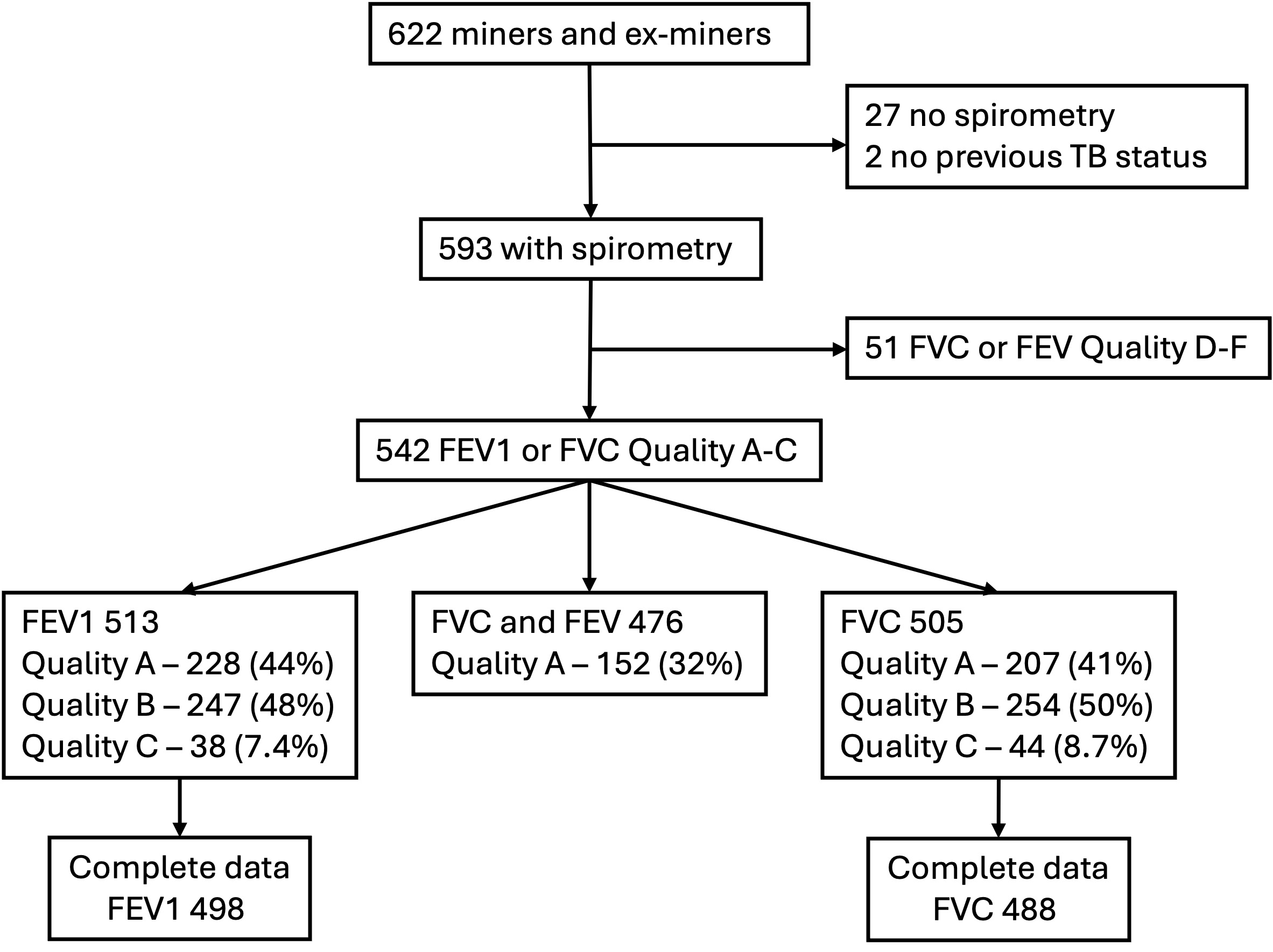


Supplementary Table 1. Comparison of manual quality assessment and as applied by spirometry software. The spirometry software also used the 2019 ATS/ERS criteria, however applied only a single score A-U. Manual scoring gave a separate score to FEV and FVC and reviewed flow loops. The table below includes all spirometry readings. Among spirometry readings from the software with a D or greater score 13 readings were upgraded to A to C values for *both* FEV1 and FVC during the manual reading, while 4 were downgraded from software A to C readings, to manual D or greater readings for *both* FEV1 and FVC.

|  | Spirometry software scoring (ATS/ERS 2019 – single score) | | | | |  |
| --- | --- | --- | --- | --- | --- | --- |
| Manual scoring (ATS/ERS separate scoring) | A (N=286) | B (N=113) | C (N=70) | D (N=116) | >D  (N=8) | Total (N=593) |
| **FVC** |  |  |  |  |  |  |
| A | 159 (56%) | 38 (34%) | 6 (9%) | 4 (3%) | 0 (0%) | 207 (35%) |
| B | 126 (44%) | 73 (65%) | 31 (44%) | 21 (18%) | 3 (38%) | 254 (43%) |
| C | 0 (0%) | 1 (1%) | 31 (44%) | 12 (10%) | 0 (0%) | 44 (7%) |
| D | 0 (0%) | 0 (0%) | 1 (1%) | 24 (21%) | 0 (0%) | 25 (4%) |
| E | 0 (0%) | 1 (1%) | 1 (1%) | 39 (34%) | 3 (38%) | 44 (7%) |
| F | 1 (0%) | 0 (0%) | 0 (0%) | 10 (9%) | 2 (25%) | 13 (2%) |
| U | 0 (0%) | 0 (0%) | 0 (0%) | 6 (5%) | 0 (0%) | 6 (1%) |
| **FEV1** |  |  |  |  |  |  |
| A | 170 (59%) | 42 (37%) | 11 (16%) | 5 (4%) | 0 (0%) | 228 (38%) |
| B | 116 (41%) | 68 (60%) | 38 (54%) | 22 (19%) | 3 (38%) | 247 (42%) |
| C | 0 (0%) | 2 (2%) | 20 (29%) | 14 (12%) | 2 (25%) | 38 (6%) |
| D | 0 (0%) | 1 (1%) | 1 (1%) | 26 (22%) | 0 (0%) | 28 (5%) |
| E | 0 (0%) | 0 (0%) | 0 (0%) | 36 (31%) | 1 (12%) | 37 (6%) |
| F | 0 (0%) | 0 (0%) | 0 (0%) | 8 (7%) | 2 (25%) | 10 (2%) |
| U | 0 (0%) | 0 (0%) | 0 (0%) | 5 (4%) | 0 (0%) | 5 (1%) |

# **Supplementary Table 2**. Table of symptoms among miners and ex-miners attending the Occupational Health Screening Centre at KIDH, Tanzania, according to previous TB status

Chi^2^ test is used for comparison

|  | Previous TB (N=80) | No Previous TB (N=462) | All participants (N=542) | p value |
| --- | --- | --- | --- | --- |
| **Cough (any duration)** |  |  |  | 0.254 |
| No | 29 (36.2%) | 138 (29.9%) | 167 (30.8%) |  |
| Yes | 51 (63.8%) | 324 (70.1%) | 375 (69.2%) |  |
| N-Miss | 0 | 0 | 0 |  |
| **Haemoptysis** |  |  |  | 0.111 |
| No | 72 (91.1%) | 440 (95.4%) | 512 (94.8%) |  |
| Yes | 7 (8.9%) | 21 (4.6%) | 28 (5.2%) |  |
| N-Miss | 1 | 1 | 2 |  |
| **Fever** |  |  |  | 0.243 |
| No | 44 (55.0%) | 286 (61.9%) | 330 (60.9%) |  |
| Yes | 36 (45.0%) | 176 (38.1%) | 212 (39.1%) |  |
| N-Miss | 0 | 0 | 0 |  |
| **Weight loss** |  |  |  | 0.640 |
| No | 42 (52.5%) | 255 (55.3%) | 297 (54.9%) |  |
| Yes | 38 (47.5%) | 206 (44.7%) | 244 (45.1%) |  |
| N-Miss | 0 | 1 | 1 |  |
| **Night sweats** |  |  |  | 0.926 |
| No | 50 (62.5%) | 290 (63.0%) | 340 (63.0%) |  |
| Yes | 30 (37.5%) | 170 (37.0%) | 200 (37.0%) |  |
| N-Miss | 0 | 2 | 2 |  |
| **Fatigue** |  |  |  | 0.773 |
| No | 36 (45.0%) | 215 (46.7%) | 251 (46.5%) |  |
| Yes | 44 (55.0%) | 245 (53.3%) | 289 (53.5%) |  |
| N-Miss | 0 | 2 | 2 |  |
| **Loss of appetite** |  |  |  | 0.585 |
| No | 49 (61.2%) | 297 (64.4%) | 346 (64.0%) |  |
| Yes | 31 (38.8%) | 164 (35.6%) | 195 (36.0%) |  |
| N-Miss | 0 | 1 | 1 |  |
| **Chest pain** |  |  |  | 0.444 |
| No | 20 (25.0%) | 134 (29.2%) | 154 (28.6%) |  |
| Yes | 60 (75.0%) | 325 (70.8%) | 385 (71.4%) |  |
| N-Miss | 0 | 3 | 3 |  |

Supplementary Table 3. Spirometry outcomes for miners and ex-miners attending the Occupational Health Screening Centre at KIDH, Tanzania, according to previous TB status, using NHANES III and 2011 GLI reference ranges, adjusted for ethnicity.

FEV1 = Forced Expiratory Volume in one second. FVC = Forced Vital Capacity. GLI = 2011 GLI global reference values. NHANES III = Third National Health and Nutrition Examination Survey, USA. Total participants FEV1 n=513; Previous TB n=75, No previous TB n=438. Total participants FVC n=505; Previous TB n=73, No previous TB n=432. Total participants FEV1/FVC ratio and category n=478; Previous TB n=68, No previous TB n=408.

|  | Previous TB | No previous TB | All participants |
| --- | --- | --- | --- |
| **FEV1 z-score category (NHANES)** |  |  |  |
| Normal | 31 (41.3%) | 333 (76.4%) | 364 (71.2%) |
| Mild | 13 (17.3%) | 51 (11.7%) | 64 (12.5%) |
| Moderate | 20 (26.7%) | 45 (10.3%) | 65 (12.7%) |
| Severe | 11 (14.7%) | 7 (1.6%) | 18 (3.5%) |
| **Percent predicted FEV1 (%) (NHANES)** |  |  |  |
| Median (Q1, Q3) | 58 (44, 73) | 77 (65, 85) | 75 (60, 83) |
| **FVC z-score category (NHANES)** |  |  |  |
| Normal | 38 (52.1%) | 362 (84.2%) | 400 (79.5%) |
| Mild | 12 (16.4%) | 40 (9.3%) | 52 (10.3%) |
| Moderate | 19 (26.0%) | 22 (5.1%) | 41 (8.2%) |
| Severe | 4 (5.5%) | 6 (1.4%) | 10 (2.0%) |
| **Percent predicted FVC (%) (NHANES)** |  |  |  |
| Median (Q1, Q3) | 64 (51, 76) | 78 (69, 86) | 77 (66, 84) |
| **Spirometry category (NHANES)** |  |  |  |
| Normal | 351 (67.1%) | 30 (34.1%) | 381 (62.4%) |
| Obstructive | 68 (13.0%) | 17 (19.3%) | 85 (13.9%) |
| Restrictive | 69 (13.2%) | 16 (18.2%) | 85 (13.9%) |
| Mixed | 35 (6.7%) | 25 (28.4%) | 60 (9.8%) |
| **FEV1 z-score category (GLI)** |  |  |  |
| Normal | 29 (38.7%) | 327 (75.0%) | 356 (69.7%) |
| Mild | 13 (17.3%) | 57 (13.1%) | 70 (13.7%) |
| Moderate | 23 (30.7%) | 46 (10.6%) | 69 (13.5%) |
| Severe | 10 (13.3%) | 6 (1.4%) | 16 (3.1%) |
| **FVC z-score category (GLI)** |  |  |  |
| Normal | 38 (52.1%) | 357 (83.0%) | 395 (78.5%) |
| Mild | 13 (17.8%) | 43 (10.0%) | 56 (11.1%) |
| Moderate | 18 (24.7%) | 25 (5.8%) | 43 (8.5%) |
| Severe | 4 (5.5%) | 5 (1.2%) | 9 (1.8%) |
| **Spirometry category (GLI)** |  |  |  |
| Normal | 29 (42.6%) | 292 (71.6%) | 321 (67.4%) |
| Obstructive | 6 (8.8%) | 46 (11.3%) | 52 (10.9%) |
| Restrictive | 12 (17.6%) | 44 (10.8%) | 56 (11.8%) |
| Mixed | 21 (30.9%) | 26 (6.4%) | 47 (9.9%) |

Supplementary Table 4. Sensitivity analysis of Spirometry outcomes for miners and ex-miners attending the Occupational Health Screening Centre at KIDH, Tanzania, according to previous TB status, restricting to only A grade spirometry

FEV1 = Forced Expiratory Volume in one second. FVC = Forced Vital Capacity. GLIgl = GLI global reference values. NHANES III = Third National Health and Nutrition Examination Survey, USA. Total participants FEV1 n=228; Previous TB n=47, No previous TB n=181. Total participants FVC n=207; Previous TB n=34, No previous TB n=173. Total participants FEV1/FVC ratio and category n=154; Previous TB n=27, No previous TB n=127.

|  | Previous TB | No previous TB | All participants |
| --- | --- | --- | --- |
| **Measured FEV1/mls** |  |  |  |
| Median (Q1, Q3) | 1881 (1438, 2452) | 2762 (2267, 3249) | 2581 (1982, 3144) |
| **Excess loss FEV1/mls (GLIgl)** |  |  |  |
| Median (Q1, Q3) | 1380 (784, 1855) | 572 (295, 1046) | 680 (342, 1265) |
| **FEV1 z-score category (GLIgl)** |  |  |  |
| Normal | 12 (25.5%) | 117 (65.0%) | 129 (56.8%) |
| Mild | 8 (17.0%) | 30 (16.7%) | 38 (16.7%) |
| Moderate | 20 (42.6%) | 31 (17.2%) | 51 (22.5%) |
| Severe | 7 (14.9%) | 2 (1.1%) | 9 (4.0%) |
| **Measured FVC/mls** |  |  |  |
| Median (Q1, Q3) | 2904 (2250, 3619) | 3478 (3008, 3989) | 3403 (2886, 3911) |
| **Excess loss FVC/mls (GLIgl)** |  |  |  |
| Median (Q1, Q3) | 981 (498, 1705) | 569 (221, 944) | 599 (309, 1053) |
| **FVC z-score category (GLIgl)** |  |  |  |
| Normal | 16 (47.1%) | 129 (75.0%) | 145 (70.4%) |
| Mild | 7 (20.6%) | 26 (15.1%) | 33 (16.0%) |
| Moderate | 7 (20.6%) | 16 (9.3%) | 23 (11.2%) |
| Severe | 4 (11.8%) | 1 (0.6%) | 5 (2.4%) |
| **FEV1/FVC ratio** |  |  |  |
| Median (Q1, Q3) | 78 (68, 82) | 81 (76, 84) | 80 (74, 84) |
| **Spirometry category (GLIgl)** |  |  |  |
| Normal | 9 (33.3%) | 85 (66.9%) | 94 (61.0%) |
| Obstructive | 3 (11.1%) | 10 (7.9%) | 13 (8.4%) |
| Restrictive | 9 (33.3%) | 23 (18.1%) | 32 (20.8%) |
| Mixed | 6 (22.2%) | 9 (7.1%) | 15 (9.7%) |
| **Excess loss FEV1/mls (NHANES)** |  |  |  |
| Median (Q1, Q3) | 1062 (465, 1542) | 264 (-38, 651) | 355 (55, 967) |
| **Excess loss FVC/mls (NHANES)** |  |  |  |
| Median (Q1, Q3) | 632 (180, 1298) | 173 (-126, 595) | 233 (-60, 695) |

# **Supplementary Table 5**. Coefficients of variables included in the model of outcome of FEV1 with the exposure of number of years of mine work and previous TB, included as an interaction term

| **Characteristic** | **Beta (mls)** | **95% CI**^1^ | **p-value** |
| --- | --- | --- | --- |
| Years worked in mining |  |  |  |
| rcs(yrs_worked_mining, knots_fev1)yrs_worked_mining | 47 | -1.2, 96 | 0.056 |
| rcs(yrs_worked_mining, knots_fev1)yrs_worked_mining' | -144 | -394, 106 | 0.3 |
| rcs(yrs_worked_mining, knots_fev1)yrs_worked_mining'' | 256 | -219, 730 | 0.3 |
| Previous TB treatment |  |  |  |
| No | — | — |  |
| Yes | 343 | -1,314, 1,999 | 0.7 |
| Age | -7.9 | -15, -0.86 | 0.028 |
| Occupation |  |  |  |
| Current miner | — | — |  |
| Ex-miner | 101 | -41, 243 | 0.2 |
| Tobacco smoker |  |  |  |
| No | — | — |  |
| Yes | -7.8 | -159, 143 | >0.9 |
| HIV status |  |  |  |
| Negative | — | — |  |
| Positive | -29 | -316, 257 | 0.8 |
| Gender |  |  |  |
| Female | — | — |  |
| Male | -267 | -896, 363 | 0.4 |
| Years worked in mining * Previous TB treatment |  |  |  |
| rcs(yrs_worked_mining, knots_fev1)yrs_worked_mining * Yes | 52 | -215, 319 | 0.7 |
| rcs(yrs_worked_mining, knots_fev1)yrs_worked_mining' * Yes | -185 | -1,279, 910 | 0.7 |
| rcs(yrs_worked_mining, knots_fev1)yrs_worked_mining'' * Yes | 280 | -1,683, 2,243 | 0.8 |
| ^1^CI = Confidence Interval | | | |

# **Supplementary Table 6**. Coefficients of variables included in the model of outcome of FVC with the exposure of number of years of mine work and previous TB, included as an interaction term

| **Characteristic** | **Beta (mls)** | **95% CI**^1^ | **p-value** |
| --- | --- | --- | --- |
| Years worked in mining |  |  |  |
| rcs(yrs_worked_mining, knots_fvc)yrs_worked_mining | 35 | -18, 87 | 0.2 |
| rcs(yrs_worked_mining, knots_fvc)yrs_worked_mining' | -112 | -382, 158 | 0.4 |
| rcs(yrs_worked_mining, knots_fvc)yrs_worked_mining'' | 220 | -291, 732 | 0.4 |
| Previous TB treatment |  |  |  |
| No | — | — |  |
| Yes | 513 | -1,180, 2,205 | 0.6 |
| Age | -11 | -19, -3.9 | 0.003 |
| Occupation |  |  |  |
| Current miner | — | — |  |
| Ex-miner | 73 | -81, 228 | 0.4 |
| Tobacco smoker |  |  |  |
| No | — | — |  |
| Yes | -86 | -249, 78 | 0.3 |
| HIV status |  |  |  |
| Negative | — | — |  |
| Positive | -102 | -388, 184 | 0.5 |
| Gender |  |  |  |
| Female | — | — |  |
| Male | 222 | -380, 825 | 0.5 |
| Years worked in mining * Previous TB treatment |  |  |  |
| rcs(yrs_worked_mining, knots_fvc)yrs_worked_mining * Yes | 28 | -249, 305 | 0.8 |
| rcs(yrs_worked_mining, knots_fvc)yrs_worked_mining' * Yes | -89 | -1,241, 1,062 | 0.9 |
| rcs(yrs_worked_mining, knots_fvc)yrs_worked_mining'' * Yes | 92 | -1,980, 2,165 | >0.9 |
| ^1^CI = Confidence Interval | | | |

Supple
